# The technology behind TB DEPOT: a novel public analytics platform integrating tuberculosis clinical, genomic, and radiological data for visual and statistical exploration

**DOI:** 10.1093/jamia/ocaa228

**Published:** 2020-11-05

**Authors:** Alyssa Long, Alexander Glogowski, Matthew Meppiel, Lisa De Vito, Eric Engle, Michael Harris, Grace Ha, Darren Schneider, Andrei Gabrielian, Darrell E Hurt, Alex Rosenthal

**Affiliations:** Department of Health and Human Services, Office of Cyber Infrastructure and Computational Biology, National Institute of Allergy and Infectious Diseases National Institutes of Health, Bethesda, Maryland, USA

**Keywords:** clinical research informatics, tuberculosis, cohort creation, data analysis, data integration

## Abstract

**Objective:**

Clinical research informatics tools are necessary to support comprehensive studies of infectious diseases. The National Institute of Allergy and Infectious Diseases (NIAID) developed the publicly accessible Tuberculosis Data Exploration Portal (TB DEPOT) to address the complex etiology of tuberculosis (TB).

**Materials and Methods:**

TB DEPOT displays deidentified patient case data and facilitates analyses across a wide range of clinical, socioeconomic, genomic, and radiological factors. The solution is built using Amazon Web Services cloud-based infrastructure, .NET Core, Angular, Highcharts, R, PLINK, and other custom-developed services. Structured patient data, pathogen genomic variants, and medical images are integrated into the solution to allow seamless filtering across data domains.

**Results:**

Researchers can use TB DEPOT to query TB patient cases, create and save patient cohorts, and execute comparative statistical analyses on demand. The tool supports user-driven data exploration and fulfills the National Institute of Health’s Findable, Accessible, Interoperable, and Reusable (FAIR) principles.

**Discussion:**

TB DEPOT is the first tool of its kind in the field of TB research to integrate multidimensional data from TB patient cases. Its scalable and flexible architectural design has accommodated growth in the data, organizations, types of data, feature requests, and usage. Use of client-side technologies over server-side technologies and prioritizing maintenance have been important lessons learned. Future directions are dynamically prioritized and key functionality is shared through an application programming interface.

**Conclusion:**

This paper describes the platform development methodology, resulting functionality, benefits, and technical considerations of a clinical research informatics application to support increased understanding of TB.

## INTRODUCTION

Clinical research informatics tools have expanded the possibilities for clinical research discovery by offering researchers new perspectives and reproducible approaches for visualizing and analyzing heterogenous data.^1–3^ The National Institute of Allergy and Infectious Diseases (NIAID) embraces this trend by bringing software developers and researchers together to develop tools that advance scientific understanding of infectious diseases and allergic conditions. The Tuberculosis Data Exploration Portal (TB DEPOT: https://depot.tbportals.niaid.nih.gov/) is one such example that seeks to address the challenges posed by tuberculosis (TB), with an emphasis on drug-resistant tuberculosis (DR-TB).^4^ TB DEPOT is a web application that aggregates a global collection of deidentified medical records (clinical, socioeconomic, pathogen genomic, radiological) for patients with TB and allows users to view, filter, and compare groups of patients based on their medical profiles. The complex etiology and treatment of TB highlight the need for a holistic research and development approach that integrates all relevant TB data domains in a collaborative environment.^5–9^ Clinical and socioeconomic, genomics, and radiological data typically reside in domain-specific silos on existing platforms that are curated and governed by different hospitals or research institutions, presenting issues with data standardization, interoperability, and data quality.^10–12^ Many of these platforms have limited visualization and analysis capabilities for data exploration, and they require technical expertise to combine and query the data, which limits our ability to obtain a comprehensive view of TB.

## OBJECTIVE

Our aim for TB DEPOT is to create an open-access, user-friendly clinical research informatics platform to contribute to research of novel drug targets, vaccines, diagnostics, and treatment strategies for TB. The solution should allow researchers to quickly view and analyze the clinical, socioeconomic, bacterial genomic, and radiological data together in one place, thus facilitating data exploration and hypothesis testing in a statistically verified manner. In the paper “TB DEPOT (Data Exploration Portal): A multidomain TB data analysis resource,” Gabrielian et al provide the scientific rationale and example use cases for TB DEPOT.^13^ This article focuses on the technical methodology and software approaches used in its development.

TB DEPOT leverages data collected as part of NIAID’s TB Portals Program (TBPP), in which a scientific consortium of institutions contribute deidentified patient data to a repository stored using the Health Level Seven International (HL7) Fast Healthcare Interoperability Resources (FHIR) standard.^14^^,^^15^ Currently, NIAID’s TBPP contains data from more than 2900 published, curated TB cases collected across 15 countries (Azerbaijan, Belarus, China, Republic of the Congo, Georgia, India, Kazakhstan, Kyrgyzstan, Mali, Moldova, Nigeria, Romania, South Africa, South Korea, Ukraine), with an additional 2900 cases that are still in review and available only for data owners. The program has collected more than 2800 Mycobacterium tuberculosis (Mtb) genomic sequences, 1600 computerized tomography (CT) studies, and 5000 chest X-ray (CXR) images.

## MATERIALS AND METHODS

### Software development approach

The original high-level requirements for TB DEPOT include the ability to: 1) quickly create cohorts defined across clinical, socioeconomic, genomic, and radiological domains with structured and unstructured data; 2) create and save data queries; 3) conduct on-demand comparative analyses of categorical and continuous variables between defined cohorts; and 4) present user-friendly statistical results and visualizations. TB DEPOT was initially developed using a waterfall approach, and then switched to a hybrid agile approach for subsequent monthly releases. Features are defined and prioritized by NIAID and the overall TBPP consortium members on an annual basis. User stories are largely sourced and prioritized by the product owner and organized into monthly sprints. The team uses Azure DevOps Server to manage the product backlog and sprints.

During sprint cycles, the team applies human-centered design principles to optimize end-user navigation and follows best practices for development including project life cycle governance, iterative prototyping, code peer review, security, and continuous integration. Automated testing of the solution with Selenium reconciled time-intensive manual execution of significant regression test cases (eg, validation of the query builder) for all 150+ attributes in the application.

### Reference architecture

**Figure 1.**
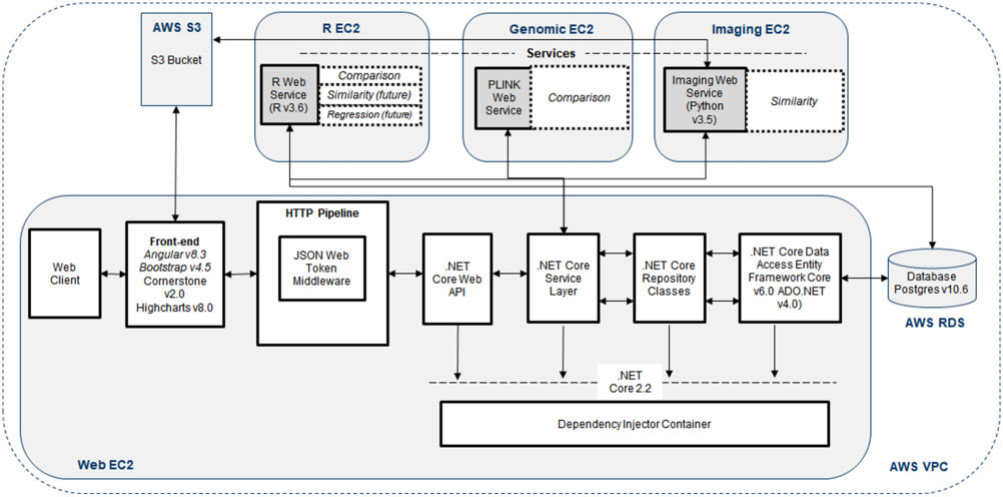
Reference architecture for TB DEPOT.

The rationale for selecting technologies for TB DEPOT was based on optimizing specific criteria: 1) scalability: ability to accommodate a growing data store over time; 2) extensibility: ability to add and update functionality; 3) complexity of integration and implementation: time and level of effort required; 4) cost considerations; and 5) global application performance: response time of the site. The solution reference architecture for TB DEPOT is depicted in Figure 1 and explained in detail below. 

### Front-end and HTTP pipeline

TB DEPOT is a web application with the front-end built on Angular v8.3 and Bootstrap v4.5, utilizing Highcharts v8.0 to display visualizations and Cornerstone v2.0 (a JavaScript based open-source viewer) to display DICOM images, which are sourced from an AWS S3 bucket. Angular’s HttpClientModule is leveraged for HTTP requests to the application programming interface (API).

### .NET solution

The .NET solution runs on .NET Core v2.2 (an open-source development platform maintained by Microsoft), and encompasses the components from the web API to the entity framework and database interaction. The Service Layer is where the application business logic resides. It manages requests between the data, repository classes, and framework via Microsoft .NET and web services. It also interacts with the R, genomic, and imaging similarity web services, which are each hosted on separate AWS Elastic Compute Cloud (EC2) instances. The R web service calls R v3.6 when the user runs a cohort comparison and, in an upcoming release, will include patient case similarity and cohort regression. For analysis of genomic variance, a genome-wide association study (GWAS) web service identifies statistically significant single nucleotide polymorphisms (SNPs) between cohorts using PLINK v1.9, an open-source whole genomic association analysis toolset.^16^^,^^17^ The Imaging web service runs on Linux and is called when the user selects the “Find Similar” feature to retrieve similar images. The Repository Classes serve as the data layer that tightly maps to the database. Entity Framework Core v6.0 is an open-source and cross-platform version of the popular Entity Framework data access technology by Microsoft. It serves as an object-relational mapper, enabling the solution to work with a database using .NET objects and eliminating the need for most of the data-access code. The API layer acts as an endpoint to the service layer. It authenticates users via a custom one-time PIN service and generates a signed JavaScript Object Notation (JSON) web token, which is subsequently used in all API requests for authorization.

### AWS infrastructure

All solution components are hosted in Amazon Web Services (AWS), which allows administrators to easily scale and add resources to support application features and data storage needs, allocate resources while minimizing costs, and maintain security. TB DEPOT’s environment resides in the us-east-1 region of AWS. It utilizes EC2 instances to run the web front-end, R, genomics, and imaging services. Each environment (Development, Quality Assurance, Production) has its own virtual private cloud that is not linked. Backups of EC2 instances are handled by scheduled Amazon Machine Image creation as well as underlying elastic block store volume backups. Security and vulnerability patching are handled by scheduled tasks on the Windows-based instances, which ensures that patches can be readily applied in development and thoroughly tested. Network security is handled by two security groups which act as stateful firewalls in AWS. The Development and Quality Assurance environments have security group rules only allowing private access to all resources. In Production, a security group with public access to ports 80 and 443 is used to allow public access to the website.

The back-end PostgreSQL database is running on Amazon’s Relational Database Service, which is a fully managed database offering, and automated nightly backups are scheduled on all databases. TB DEPOT leverages Amazon S3 for long-term object storage using private S3 buckets for each environment. Access to the AWS environment is controlled via identity and access management (IAM) users with two-factor authentication, and IAM groups and IAM roles are used to manage policies for each service.

### Solution data flow

**Figure 2.**
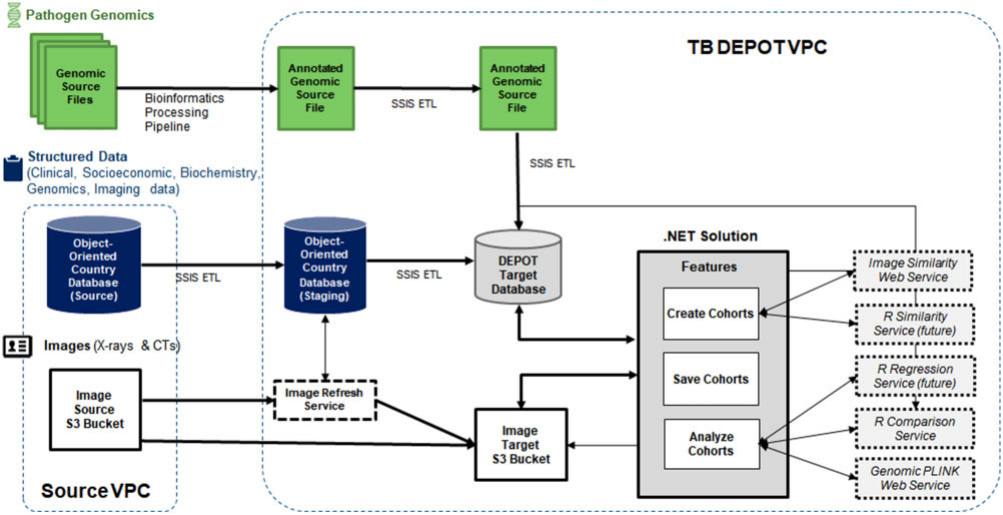
Data flow diagram for TB DEPOT.

There are three primary sources of data that are integrated into TB DEPOT as shown in Figure 2 below. 

Deidentified structured data are collected and stored in a PostgreSQL database. Data are either entered manually by TBPP consortium members or loaded by developers using an HL7 FHIR API for bulk data feeds.^10^ The clinical, socioeconomic, genomic, and imaging metadata are stored in JSON format using HL7 FHIR data exchange standard for data interoperability.^15^ Microsoft SQL Server Integration Services (SSIS) packages were developed to extract data from the source database to a staging area that replicates the source JSON structure. Another SSIS package extracts, transforms, and loads data from the staging JSON format to a normalized, relational data model in the target database.

Pathogen genomics source data are first annotated in a bioinformatics processing pipeline. TBPP involves several sequencing centers for full genome sequencing of Mtb.^10^ Bacterial DNA is sequenced from patient specimens, and raw sequence reads are analyzed and converted into binary vectors of SNPs characterizing resistance to known drugs. The annotated results are copied and loaded from comma-separated values files into the target database via an SSIS package.

CXR and CT images are automatically deidentified by TBPP and stored in AWS S3. All CT studies and most CXRs follow the Digital Imaging and Communications in Medicine (DICOM) standard. When DICOM CXRs are not available, analog CXRs are captured using a digital camera or scanner from film radiographs. To keep the images synchronized with the data, a custom image refresh service is used to sync images from the source S3 bucket to the target S3 bucket. CXRs and CT studies can be viewed through the integrated CT image viewer, Cornerstone.^18^ Users can download the raw images after completing a data use agreement.

All data additions and modifications are made using the truncate and load methodology and logged in audit tables, allowing for reproducibility of datasets. Structured data are joined to unstructured data by storing the genomic Sequence Read Archive links for each sequence and imaging S3 bucket file paths for each image. Structured data and images are updated weekly via a SQL Job Agent and Windows Task Scheduler, while pathogen genomic data are added ad hoc as sequences are annotated in batches. The solution uses service accounts and Secure Sockets Layer, a standard security protocol, to ensure all data are encrypted while in motion for data security. Once the data-loading process is complete, views and functions are created to support the .NET solution Features and web services, which are described further in the Results section.

**Figure 3.**
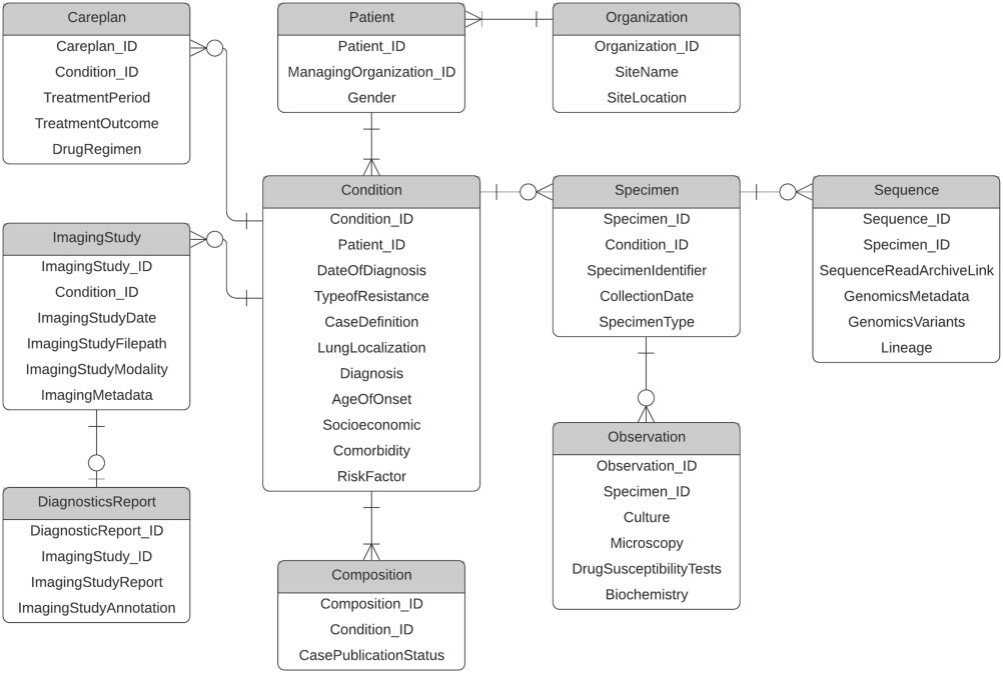
Simplified version of entity relationship diagram for target database.

A simplified version of the Entity Relationship Diagram in Figure 3 depicts the structured patient case-centric source data domains and their associated subdomains and relationships. Clinical and socioeconomic domains capture patient medical history, socioeconomic status, specimens, and lab test results. The genomic domain contains bacterial genomes from patient specimens, primarily sputum. The imaging domain captures metadata about the images and clinical annotations. All data ties back to the patient case via the Condition table. 

Patient cases are not visible to the public, or “published”, until they have been verified and approved by a consortium member. Domain coverages for published cases vary with clinical being the most comprehensive at 100% having clinical data, followed by 69% having CXR or CT images, and 64% having bacterial genomes. 36% of current cases have clinical, genomic, and imaging data. Besides the hospital or organization where the patient data are collected, spatial and epidemiological variables are not captured.

## RESULTS

**Figure 4.**
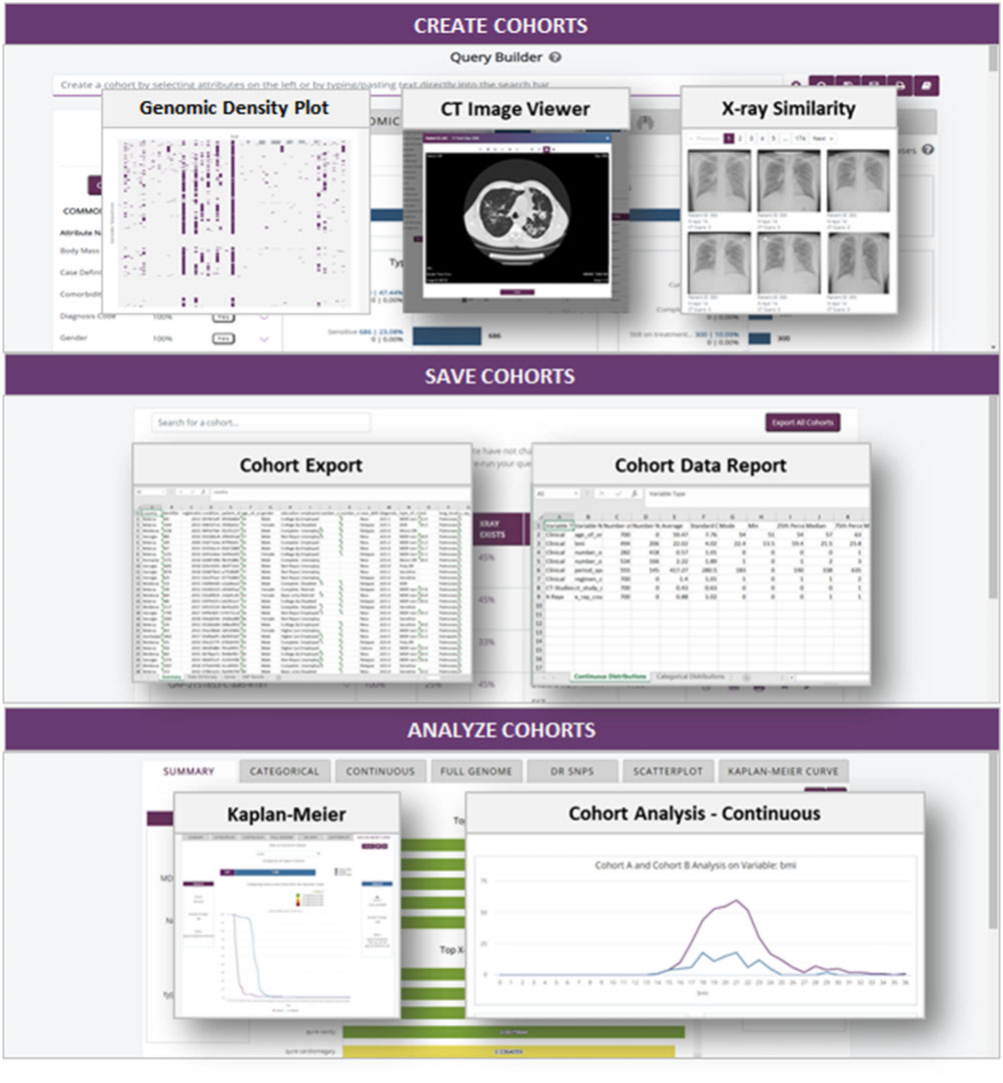
Sample screenshots of TB DEPOT user interface.

TB DEPOT was designed to fulfill the National Institute of Health’s Findable, Accessible, Interoperable, and Reusable (FAIR) principles.^19^ TB DEPOT allows users to explore the data through creating, saving, and analyzing cohorts, as shown in Figure 4. Users can begin a query in one domain, Clinical, Genomics, CT, or X-ray and traverse across all four domains to create cohorts. Users can save and rerun cohorts at any time for reproducibility and run comparative analyses to explore differences between two cohorts. 

Visualizations and statistical tests as pictured in Figure 4 are specifically designed using human-centered design principles to allow users to easily navigate between domains and conduct analyses that would otherwise require a trained data scientist. The user interface was iteratively shaped by user personas, workflows, wireframes, and site page composites for targeted user groups of clinical researchers, bioinformaticians, and radiologists. As denoted in the Data Flow Diagram and Sample Screenshots, application features are organized as: 1) Create Cohorts, 2) Save Cohorts, and 3) Analyze Cohorts. Note that the functionality highlighted does not represent an exhaustive list but previews the activities that users will engage in as part of their analyses.

### Cohort creation

Each tab within cohort creation displays the total count of patient cases in the selected cohort with distributions for key characteristics. The Query Builder displays the filters applied as users build queries using Boolean operators, and each filter attribute and its values are defined in the data dictionary. The logic for the Query Builder is built using C#, JavaScript, jQuery, SQL, and HTML. Once a user submits a query, this action triggers an AJAX request to the .NET business logic that parses the input via a custom formatter, replaces display-formatted attributes with database values, parses the Boolean logic operators and nested queries to convert into database query language, and generates an SQL query to run against the database. The business logic sends the list of cases back to the JavaScript call to filter the page visualizations.

As users execute queries against the population of patient cases, Highcharts visualizations are updated in real time to show the impact of each filter. Data domains are split up into four distinct tabs described in Table 1. 

**Table 1.** Cohort creation user interface organization

Data Domain	# of Filters	Description
Clinical	140 attributes	Clinical and socioeconomic data distributions for key characteristics are presented as cards that can be dynamically added and rearranged
Genomics	10 pathogen genomic annotations	SNP vector variants of DR-TB strains are visualized using density maps. A hierarchical cluster analysis is precomputed in R on all SNP vectors to sort sequences based on genomic profiles. Density maps display similar groupings of genomic SNP variants by drug-resistance associations, genes, and individual SNPs
CT Studies	23 manual annotations	All CT studies are viewable in Cornerstone and each series can be played by instance, allowing users to visually inspect and interact with the imaging study
X-rays	29 manual annotations + 12 automated annotations	All CXRs are viewable in Cornerstone. Users can also upload their own CXR or choose an existing CXR to find the most similar images through the imaging similarity web service

Abbreviations: CT, computerized tomography; CXR, chest X-ray; DR-TB, drug-resistant tuberculosis; SNP, single nucleotide polymorphisms.

CXRs have two types of annotations available. Manual annotations are recorded by a radiologist or pulmonologist. Automated annotations are generated by a deep learning algorithm provided by program collaborator QureAI.^20^^,^^21^

### Saved cohorts

Once a user creates a cohort of interest, they can save it for subsequent analyses or export. Saved cohort metadata includes the user-generated cohort name, creation timestamp, and case count. Users can view saved cohorts as is to reproduce results, analyze two cohorts, rename, delete, or rerun the query after data refreshes. If a user wants to export cohort data, they can request collaboration through the application, whereupon TBPP will review the user’s justification and ask them to sign a data use agreement. Cohort data exports return a spreadsheet with raw data, genomic variants, and data dictionary. The API component in Figure 1 is used to create, update, and retrieve cohorts, which can be used across all TBPP tools.

### Cohort analysis

The cohort analysis comparison feature compares two saved cohorts across all data domains. Cohort analysis is designed to compare groups with minimal overlap; therefore, cases are excluded from the statistical analysis if they exist in both cohorts.

### Statistical methods

An analysis of distributions for each attribute is conducted through real-time calls to the R Web Service (R v3.6.0). Categorical attributes are assessed using Fisher’s exact test, while continuous attributes are assessed using a Mann-Whitney U Test. For categorical variables, the resulting *P* value from Fisher’s exact test assesses the null hypothesis that the cohorts are identical. Fisher’s exact test is preferred over a Chi-squared test since the latter uses a distribution that may not hold for small cohort samples.^22^ The solution uses the fisher.test function in the R stats package.^23^ For large cohorts when the list of values is too computationally expensive, the function runs a Monte Carlo simulation to achieve a more accurate estimate of the *P* value. To mitigate performance costs, we partition the categorical variable set into two cores and run the function in parallel using the R parallel package. For continuous variables, the *P* value indicates the probability that a randomly selected value from one sample is less than or greater than a randomly selected value from a second sample. To perform the unpaired two-samples Mann-Whitney U test, the calculation is implemented in R using the wilcox.test function in the stats package.^24^

A Kaplan-Meier curve shows probability of an outcome at a certain time interval. Survival curves of the two cohorts are compared using a log-rank test to generate a *P* value from a Chi-squared distribution. These calculations are performed using the R survival package.^25^

### GWAS analysis

To identify genomic sequences with significantly different SNPs between the compared cohorts, the authors developed a web service to transfer SNP data to the PLINK v1.9 whole genome association analysis tool.^16^^,^^17^ PLINK is an open-source tool used to analyze raw variant call format files. PLINK analyzes all variants in the genomes, not just SNPs already known to be associated with Mtb resistance. The PLINK web service is implemented through JAX-RS (REST Web services) 2.0 and hosted via Tomcat.

### Cohort analysis results

Cohort comparison statistical analysis results are written back to the database and presented along with visualizations developed in Highcharts in the tabs listed in Table 2. 

**Table 2.** Cohort analysis user interface organization

Feature	Description
Summary of Asymmetries	Attributes presented in descending order of significance for each group: clinical, genomic, CT, X-ray, and GWAS asymmetries. Ranking is based on uneven distribution characterized by *P* values.
Categorical	Bar charts and block charts display differences between 2 cohorts for the user-selected categorical variable. Displays distributions and expected values from the Fisher’s exact test.
Continuous	Line graphs and box and whisker plots display differences between 2 cohorts for the user-selected continuous variable. Displays distributions and expected values from the Mann-Whitney U test.
Full Genome	PLINK results of GWAS analysis are presented as a scatterplot against the corresponding genomic position. A tabular view shows the gene, amino acid change, genomic position, *P* value, sample size in each cohort, impact of the amino acid change, and associated protein information
DR SNPs	Displays a subset of SNPs linked to drug resistance. A summary-level bar chart showing the distribution and difference in percentage of known mutations for each drug, gene, and variant, followed by an SNP density plot for each cohort.
Scatterplot	Plot any categorical variable on the x-axis and any continuous variable on the y-axis. Cohorts are plotted with different shapes and colors to represent differences.
Kaplan-Meier	Time-to-event curves are plotted for each cohort, with the start of treatment normalized as day zero. The difference between 2 cohorts is plotted with a corresponding *P* value for selected outcome.

Abbreviations: CT, computerized tomography; CXR, chest X-ray; DR, drug-resistant; GWAS, genome-wide association study; SNP, single nucleotide polymorphisms.

## DISCUSSION

TB DEPOT is the first analytics platform of its kind in the field of TB research to integrate multidimensional clinical, socioeconomic, genomic, and imaging data from TB patient cases. During its development and successive version releases, the developers encountered and addressed a variety of technical and user experience considerations. Here, we discuss the project successes to date, lessons learned, and future directions.

### Project successes

Since the first release of TB DEPOT, its scalable and flexible architectural design has accommodated growth in the data, organizations in the consortium, types of data, feature requests, and usage. As the number of cases, genomic sequences, and images have increased, the resources in AWS can easily be scaled and load-balanced to address rising storage and performance needs.^26^^,^^27^ As new organizations join the consortium, the standardized data collection in HL7 FHIR and self-service analysis capabilities in TB DEPOT have simplified the onboarding process. When new data types or new features are requested, such as an addition of biochemistry data or the concept of domain similarity, the modular design allows each developer to work on updates to specific components of the solution. The unified development process and organized team communication helps manage and streamline integration of the changes. Since TB DEPOT converted to Angular on November 25, 2019, Google Analytics tracked usage through August 24, 2020, with a brief lapse from March 13 to April 6, 2020 when Google Analytics was disabled. During this period of 249 days, TB DEPOT hosted 2350 sessions for 1182 users from 49 countries. The bounce rate was 35.1% with average session duration of 2 minutes 46 seconds for new users and almost 5 minutes for returning users. Since initial deployment, 136 external users have registered and logged into TB DEPOT, with 36 of those users logging in 3 or more times. Most importantly, users are required to sign a data use agreement to download data for their own research, and 41% of the 29 current data use agreements originated in TB DEPOT. The tool has also been used in scientific publications to advance clinical research on DR-TB.^13^^,^^20^^,^^28^^,^^29^

### Lessons learned

After the first release, the ability to consistently access the Cohort Analysis results was a challenge for international users with limited bandwidth. Originally, TB DEPOT applied a commercial-off-the-shelf (COTS) business intelligence tool to display Cohort Analysis results. The tool’s visualization objects were rendered server-side and transmitted, via hundreds of GET and POST requests, to the client browser every time an analysis was run. Users did not observe performance issues in the eastern United States where the AWS application is hosted, but the size and complexity of visualizations returned by the server led to significant latency and frequent failure in other regions. The COTS product used to produce the visualizations had also influenced other choices in the solution architecture. For example, TB DEPOT originally used ASP.NET Web Forms since the objects required a server-side framework, which relies on event-driven programming. Performance issues led to significant changes of the technical architecture in subsequent releases to shift to client-side rendering over server-side rendering. Highcharts was chosen as an open-source visualization tool to replace the COTS product, largely due to ease of development and library support. The web application framework was changed from ASP .NET Web Forms to Angular, a client-side framework, and the team is in the process of shifting automated testing efforts from Selenium to native e2e (end-to-end) test suites for both regression and iterative functional testing. After making these changes, users saw a 75% improvement on average for Cohort Analysis results return times.

Historically, the team prioritized new functionality and data collection efforts over operations and maintenance, which led to technical debt as the application grew. The team now addresses operations and maintenance activities in every release such as library and version upgrades, optimization, and scaling because the solution is stable and has a substantial amount of DR-TB patient data and a growing user base. For example, R and GWAS computations are quick (only a few seconds), so there has not previously been a need for load balancing of simultaneous calls. However, the team is now designing a First-In First-Out queueing approach for R to prevent excessive memory and CPU overload along with setting up auto-scaling and load balancing of the R and Genomics EC2 instances.

### Future directions

As the TBPP has evolved, incorporated new types of data, and tackled new research challenges, the feature backlog for TB DEPOT has continued to grow. The prioritization of application features for future releases are described in Gabrielian et al.^13^ Beyond integrating new data and variables, the concepts of patient case similarity for all domains and cohort regression will be added as new ways to create and analyze cohorts. Additionally, new visualizations and analyses centered around time-series data will provide another dimension for users to explore the data.

Outside of future directions for the tool itself, the team is looking to share the data and functionality of TB DEPOT with other tools via its API. Key features, such as saving and accessing user cohorts, obtaining case metadata, and generating user login codes have already been moved to the API. Other calculations, such as dynamic value counts for each variable and expected values based on the population, will be moved to the API. These features were selected due to the extensibility of their use in other TBPP applications. Moving forward, TBPP will continue to expand on this shared code base to reduce duplicative code and increase productivity.

The architecture, methods, and approaches used for TB DEPOT can be adapted to other disease areas and pathogens. In today’s world, there exists an unprecedented volume of clinical and research data, requiring a user-friendly and powerful advanced analytics platform. The development team is open to new opportunities for collaboration to share web services, code, and best practices to advance clinical research. The source code for TB DEPOT is not publicly shared at this time due to the effort required to properly prepare it for a community consumption; but as the program continues to advance, the team plans to share the code with requisite documentation.

## CONCLUSION

Through international collaborations between NIAID and its TBPP Consortium, NIAID has implemented TB DEPOT as a clinical research informatics application to address the challenge of TB. The hybrid agile development methodology allowed a lean team of developers to prioritize key features and iteratively develop TB DEPOT. TB DEPOT successfully integrates clinical, socioeconomic, genomic, and radiological TB data into an organized, curated dataset that can be mined and explored to test hypotheses for future research. The tool is used in clinical research, and results have been presented in scientific conferences and journal publications. The method of working with large datasets through creating user-defined virtual cohorts could be expanded to new diseases, new use cases, and supplement existing cohort identification tools, such as i2b2.^30^ As TB DEPOT’s dataset and platform continue to grow, the program invites users and potential collaborators to join this mission to advance our knowledge of TB.

## FUNDING

This research received no specific grant from any funding agency in the public, commercial, or not-for-profit sectors. However, this project has been funded in part with Federal funds from the National Institute of Allergy and Infectious Diseases (NIAID), National Institutes of Health (NIH), Department of Health and Human Services under contract HHSN316201300006W/HHSN27200002 to Medical Science & Computing, LLC, Inc and under contract HHSN316201200018W/75N98119F00012 to Deloitte Consulting LLP and under the US Civilian Research and Development Foundation (CDRF) Agreement No. BOB1-31120-MK-13.

## AUTHOR CONTRIBUTIONS

AlG, AL, MM, LD, MH, and DS are the development team for this work; GH is a technical writer and subject matter expert; AnG and EE are the product owners; and DH and AR are the product sponsors. All authors made substantial contributions to the work, reviewed and approved the publication, and agree to be accountable for all aspects of the work in ensuring that questions related to the accuracy or integrity of any part of the work are appropriately investigated and resolved.
